# Supplementary figures and images for: Multi-modal sleep intervention for community-dwelling people living with dementia and primary caregiver dyads with sleep disturbance: protocol of a single-arm feasibility trial
Source: PeerJ. 2023 Dec 14;11:e16543. doi: 10.7717/peerj.16543 (PMC10725664; doi:10.7717/peerj.16543)

Supplementary Material - Sample Pages from Workbook

**
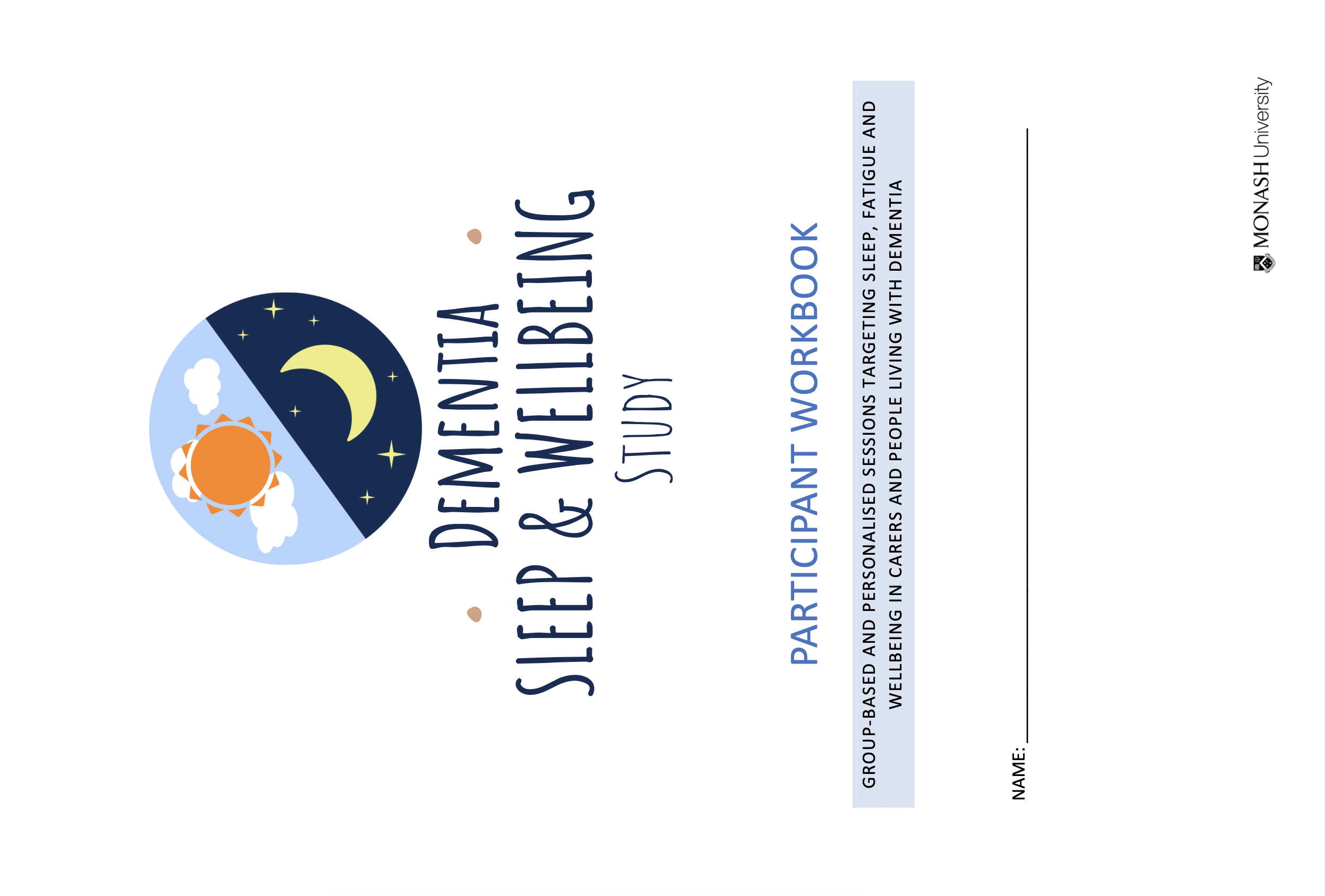
**

**
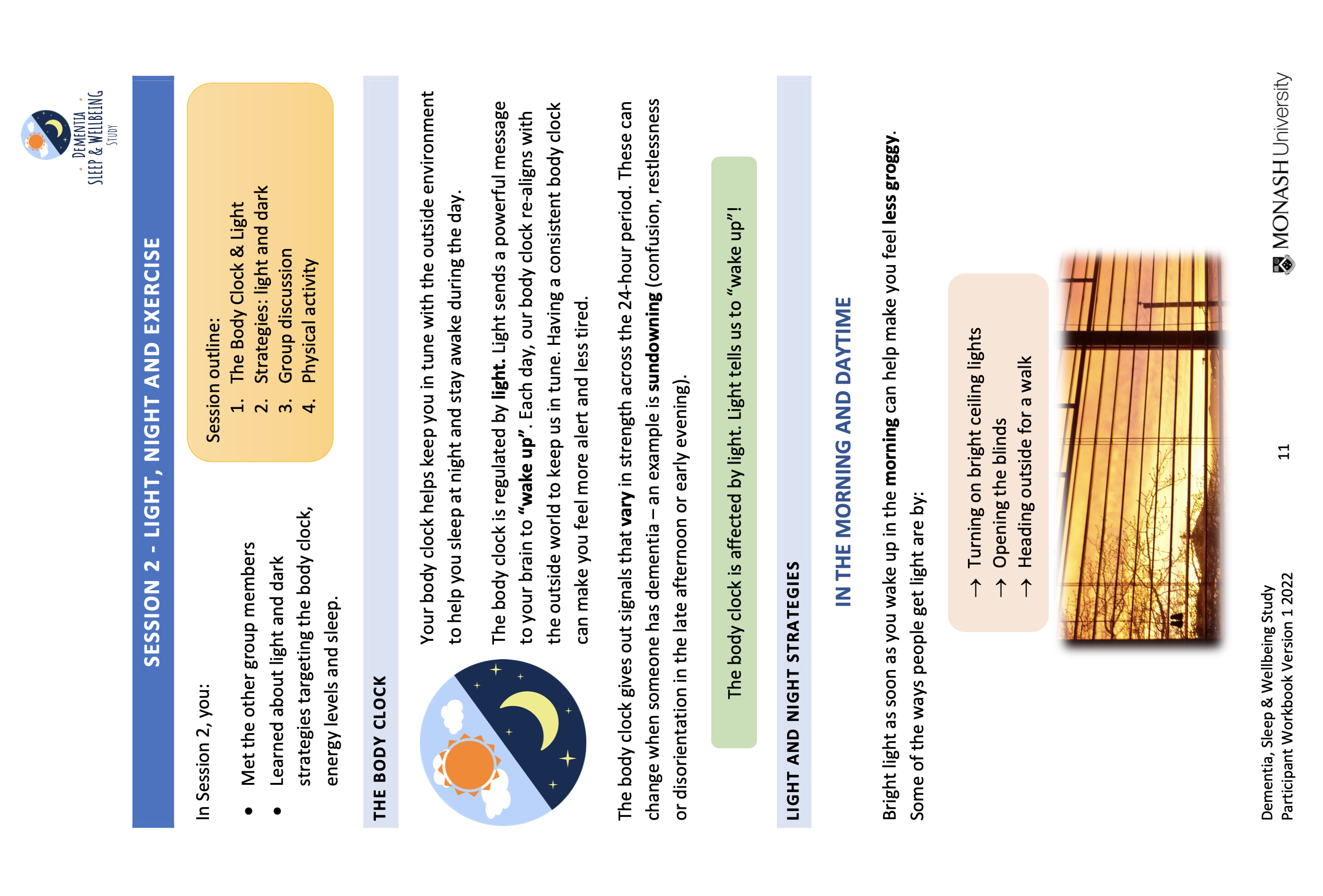
**

**
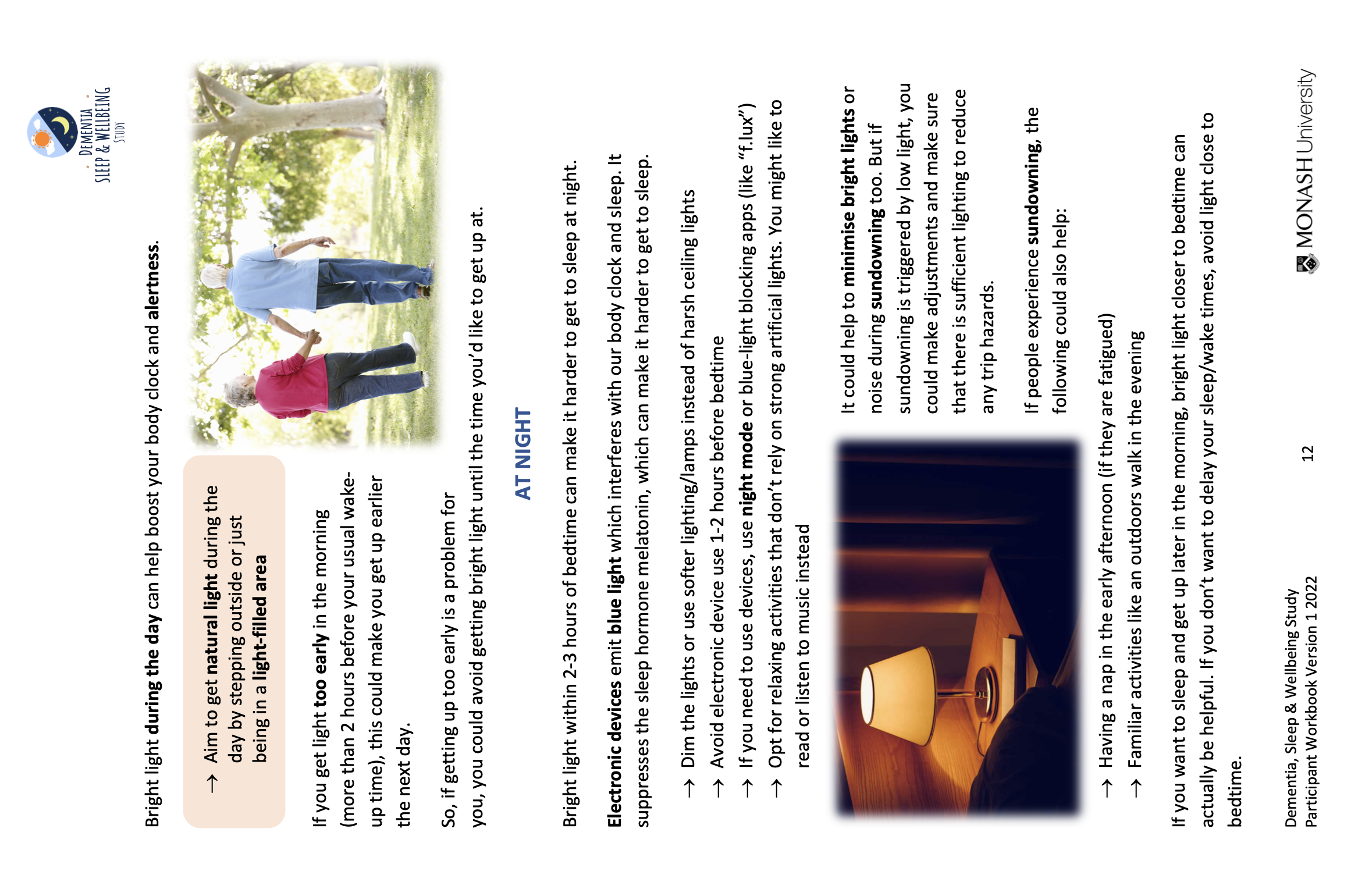
**

**
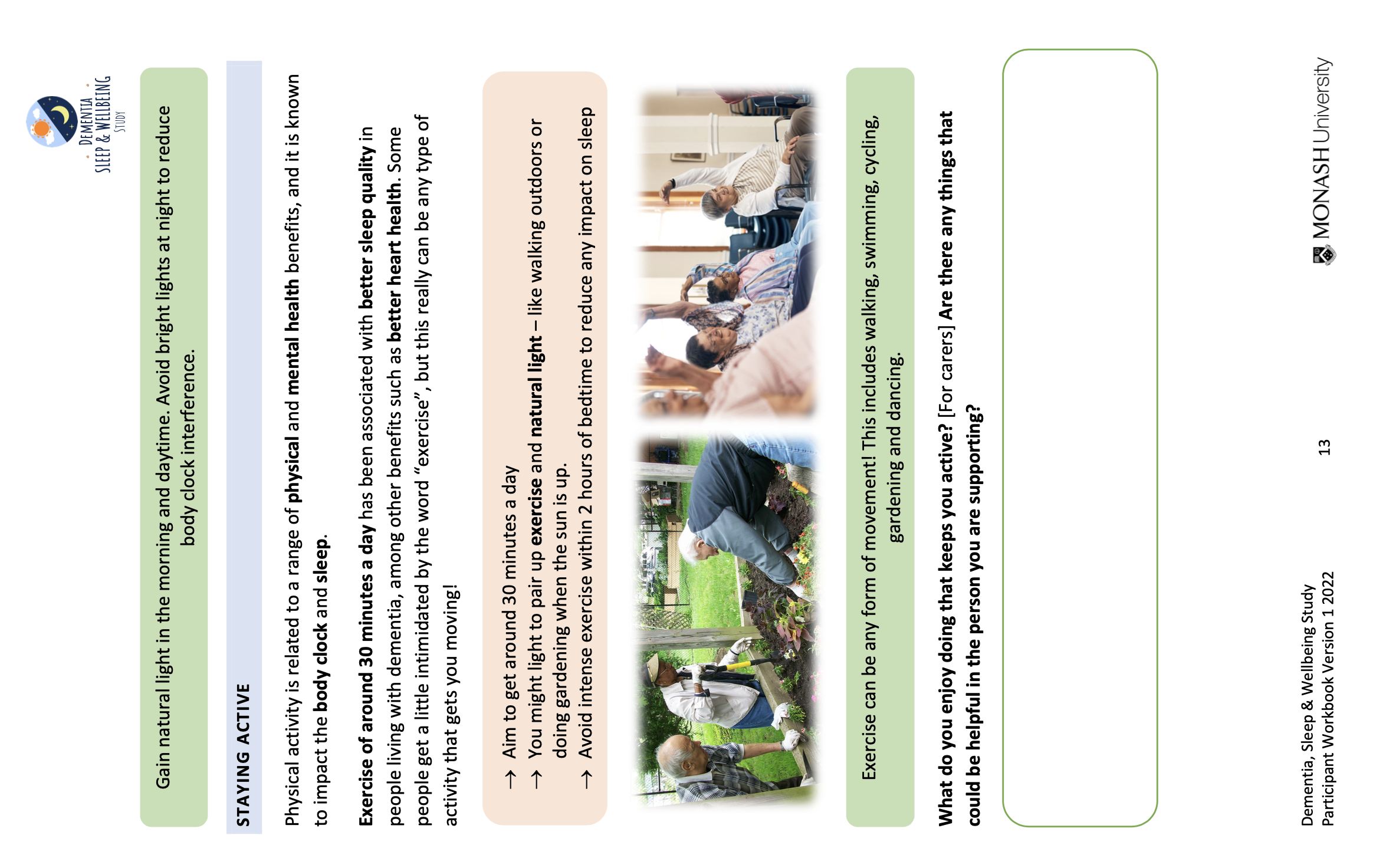
**

Supplement: Supplemental Information 3 [file peerj-11-16543-s003.docx]

Supplementary Material – Group Guidelines


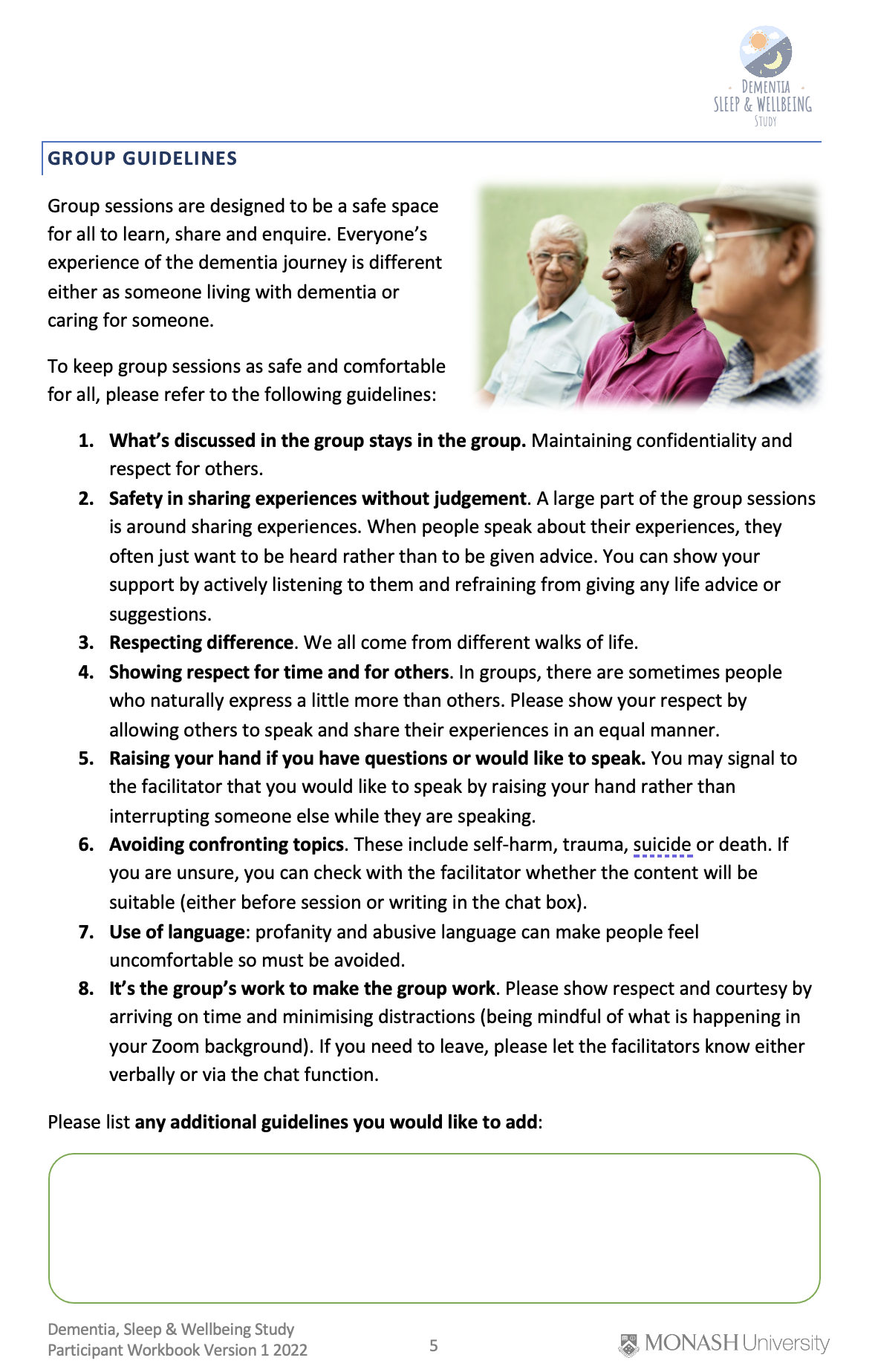

Supplement: Supplemental Information 4 [file peerj-11-16543-s004.docx]
