# Supplementary material for: Multi-modal sleep intervention for community-dwelling people living with dementia and primary caregiver dyads with sleep disturbance: protocol of a single-arm feasibility trial
Source: PeerJ. 2023 Dec 14;11:e16543. doi: 10.7717/peerj.16543 (PMC10725664; doi:10.7717/peerj.16543)
Supplement: Supplemental Information 5 [file peerj-11-16543-s005.docx]

Supplementary Material – Consent Form


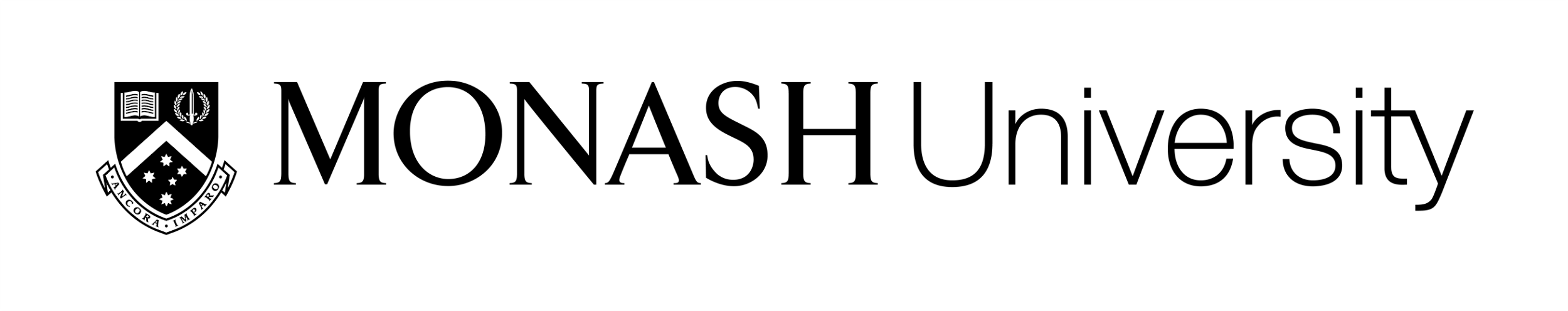


**CONSENT FORM**

**Dementia, Sleep & Wellbeing Study**

- I have read the Information Sheet or someone else has read it to me, and I have understood it fully.
- I am freely providing my consent to participate in this study.
- I agree to undergo a 6-week sleep program and complete all study procedures outlined in the information sheet.
- My participation is voluntary (my choice) and I am free to withdraw from the study at any time which will in no way affect continued treatment and support.
- I understand that the sessions will be group-based and agree to the following group session guidelines:
- Being respectful of time and of people, including allowing time for people to speak and share their experiences and opinions.
- Understanding that discussions within the group setting will remain confidential.
- While the responses from the research project will be published, I will not be identified whatsoever, and my personal details will be held strictly confidential by the research team.
- I have understood the potential risks of participating and know that I can contact the research team and other support services as per the Information Sheet if I would like additional support.
- I understand that if I wish to withdraw my consent, my de-identified responses may not be able to be deleted.
- If I have any questions, feedback or complaints about participation, I understand that I may contact the researchers or Monash University Human Research and Ethics Committee through the details below.
- I consent for the sessions to be recorded over Zoom.
- I provide my consent for members of the research team to attend the sleep program sessions.
- I understand that the information I provide can be used for this project and for future studies, and any new researchers will only have access to data that cannot identify me.

I *(print full name) ______________________________* hereby agree to the above statements and consent to take part in this study.

**I am (please tick one):**

□ a care partner

□ a person living with dementia

**Your signature**: ….............................................................

**Date**: …........................................................................................

If you would like to receive a brief summary of the results when available, please select the option below:

□ I would like to receive a copy of the results

**Email address**: ….....................................................................................

**Date: …………………………………..**

**RESEARCHERS:**

**Dr Melinda Jackson** (she/her)

*Senior Lecturer and Psychologist*

Email: melinda.jackson@monash.edu

Phone number: (03) 9905 0206

**Dr Sumedha Verma** (she/her)

*Postdoctoral Research Fellow*

Email: sumedha.verma@monash.edu

**Dr Prerna Varma** (she/her)

*Research Fellow*

Email: prerna.varma@monash.edu

**Ms Aimee Brown** (she/her)

*Research Assistant*

Email: aimee.brown@monash.edu

**Mr Chun (Arthur) Leung** (he/him)

*Honours Student*

Email: cleu0019@student.monash.edu

**ETHICS:**

**Monash University Human Research Ethics Committee (MUHREC)**

Email: muhrec@monash.edu

Phone number: (03) 9905 2052
